# Epidermal growth factor receptor levels are reduced in mice with targeted disruption of the protein kinase A catalytic subunit

**DOI:** 10.1186/1471-2121-9-16

**Published:** 2008-04-01

**Authors:** Morten P Oksvold, Ane Funderud, Anne-Katrine Kvissel, Ellen Skarpen, Heidi Henanger, Henrik S Huitfeldt, Bjørn S Skålhegg, Sigurd Ørstavik

**Affiliations:** 1 Institute of Pathology, Rikshospitalet University Hospital, University of Oslo, Norway; 2 Institute for Basic Medical Sciences, Department of Nutrition, University of Oslo Medical School, Norway

## Abstract

**Background:**

Epidermal Growth Factor Receptor (EGFR) is a key target molecule in current treatment of several neoplastic diseases. Hence, in order to develop and improve current drugs targeting EGFR signalling, an accurate understanding of how this signalling pathway is regulated is required. It has recently been demonstrated that inhibition of cAMP-dependent protein kinase (PKA) induces a ligand-independent internalization of EGFR. Cyclic-AMP-dependent protein kinase consists of a regulatory dimer bound to two catalytic subunits.

**Results:**

We have investigated the effect on EGFR levels after ablating the two catalytic subunits, Cα and Cβ in two different models. The first model used targeted disruption of either Cα or Cβ in mice whereas the second model used Cα and Cβ RNA interference in HeLa cells. In both models we observed a significant reduction of EGFR expression at the protein but not mRNA level.

**Conclusion:**

Our results suggest that PKA may represent a target that when manipulated can maintain EGFR protein levels at the single cell level as well as in intact animals.

## Background

Ligand binding to EGFR induces tyrosine transphosphorylation, and phosphotyrosines serve as binding sites for various signalling molecules. Association of these molecules with the EGFR leads to their activation, and initiation of signalling cascades culminating in a variety of responses. The activated EGFR is internalized shortly after ligand binding, and is processed in the endosomal pathway. The receptor is signalling competent when residing in the plasma membrane [1], but also during intracellular receptor trafficking [2,3]. Defects in the internalization process and degradation pathways for the EGFR family members have been associated with cell transformation and oncogenesis [4]. It has been demonstrated that cAMP-dependent protein kinase (PKA) is involved in the transduction of mitogenic signals [5], and interactions between PKA and the activated EGFR have been demonstrated [6]. Previous studies have shown that the EGFR is a substrate for PKA [7,8]. Phosphorylation of the EGFR by PKA on serine residues leads to decreased tyrosine kinase activity and diminished autophosphorylation of the EGFR [9]. Recently, Salazar and Gonzalez [10] showed that PKA basal activity controls EGFR function, both at the cell surface and during down-regulation.

PKA is a holoenzyme consisting of two regulatory (R) subunits bound together in a dimer, with one catalytic (C) subunit bound to each R-subunit [11]. In the absence of cAMP, the R-subunits will inhibit the C subunits, but a conformational change in the R-subunit is induced by binding of cAMP, releasing the C subunit which is then active. In mammals, four genes encode different isoforms of the R-subunits, RIα, RIβ, RIIα and RIIβ, and three different genes encode three isoforms of C, Cα, Cβ and PRKX [12]. The Cα and Cβ isoforms are closely related in protein sequence, whereas the PRKX sequence is divergent from Cα and Cβ. The Cα and the Cβ genes encode tissue-specifically expressed splice variants designated Cα1, Cα2, Cβ1, Cβ2, Cβ3, Cβ4, and several Cβ3 and 4abc variants [13-20].

Functional features associated with the various C subunits have been studied in genetically null mutated mice. Mutation of the Cβ gene does not result in any clear phenotype and the mice appear healthy and fertile [21]. By contrast, mutation of the Cα gene leads to early postnatal lethality in the majority of the offsprings [22]. The male Cα KO mice that survive to adulthood are infertile and both male and females show a uniform reduction in size by approximately 30% compared to their wild type littermates. Size reduction is accompanied by a nearly complete absence of PKA C subunit activity in most tissues, except the brain, where C subunit activity is slightly elevated due to Cβ compensation. Moreover, growth retardation in the Cα KO mice may be growth hormone (GH)-dependent because mRNA levels of GH-dependent molecules such as IGF-1 (insulin like growth factor 1) and MUPs (major urinary protein) were significantly reduced.

The cAMP/PKA signaling pathway may be activated through stimulation of a number of different receptors that regulate a vast number of cellular processes. These include metabolism, gene expression, ion channel conductivity, cell growth and division as well as cell differentiation [23,24]. Since the significance of PKA-dependent interaction with the EGFR is poorly understood, we embarked on a study to investigate the location and levels of EGFR in PKA C subunit null mutated mice. Our results indicate that the level and localization of EGFR are closely correlated with the level and activities of PKA C subunit.

## Results

### EGFR levels in mice are regulated by PKA catalytic subunits

**Figure 1:**
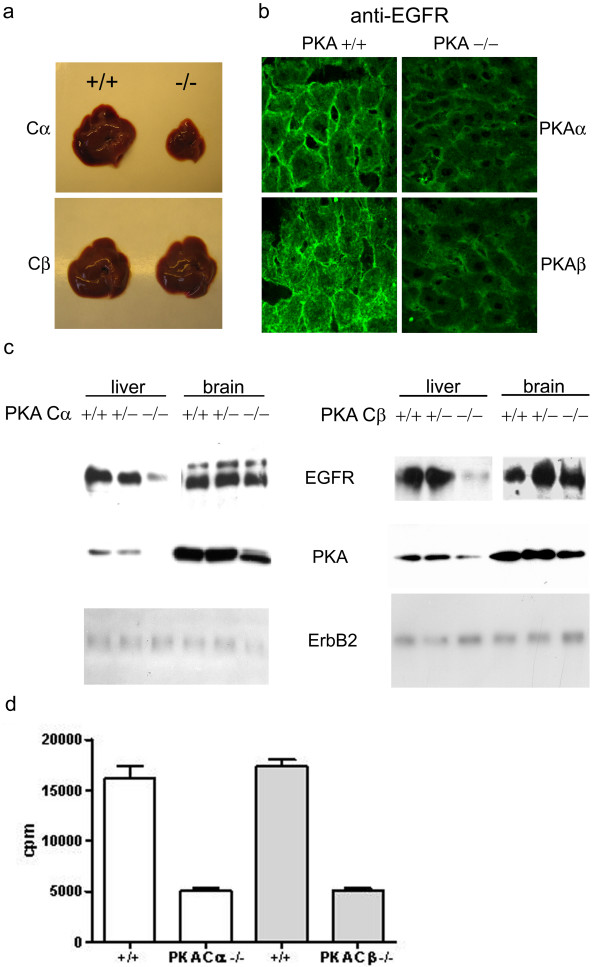
**Effect of PKA Cα or Cβ ablation on EGFR expression.** (a) Comparison of liver from wild type and PKA Cα and Cβ ablated mice. PKA Cα KO mice showed a clear uniform reduction in size. (b) Confocal immunofluorescence microscopy of frozen liver sections stained by sheep anti-EGFR and Cy3-conjugated donkey anti-sheep antibodies. (c) Western immunoblotting analysis of EGFR expression in liver and brain from wt (+/+), heterozygote (-/+), and Cα and Cβ KO (-/-) mice. Immunoblots were incubated with sheep anti-EGFR, rabbit anti-pan PKA C and mouse anti-PKA C, and rabbit anti-erbB2. Secondary HRP-conjugated anti-IgG antibodies were used for detection. (d) PKA kinase activity in mouse liver. Activity was assayed by phosphorylation of the PKA-specific substrate Kemptide using γ-[^32^P]ATP. The assay was performed in the presence of cAMP. Activity was measured by liquid scintillation in 3 ml Opti-fluor. Values are given as counts per minute (cpm).

EGFR trafficking has been demonstrated to be regulated by PKA activity, and inhibition of PKA activity results in internalization of the EGFR in neuroblastoma N2a cells [10]. We therefore decided to examine the localization of EGFR in the livers of mice depleted of either PKA Cα or Cβ [22,21]. Livers from wt and KO Cα and Cβ mice were dissected and the morphology and size examined. As illustrated in Figure 1A, livers from Cα KO mice were smaller compared to wt mice. Frozen sections of liver from wt and KO of Cα and Cβ were stained using anti-EGFR and visualized using confocal immunofluorescence microscopy. We observed a reduction in membrane association as compared to the wild-type littermates for both Cα KO and Cβ KO (Figure 1B). This led us to believe that abolished expression of either Cα or Cβ led to a decrease in total EGFR levels in liver.

To test this hypothesis, cell extracts were prepared from liver and brain of Cα and Cβ KO animals and wt and heterozygous littermates, separated by SDS-PAGE for analysis by Western immunoblotting using anti-EGFR (Figure 1C, upper panel). In liver but not brain, a clear reduction in total EGFR could be observed in both Cα KO and Cβ KO animals, as compared to their wild-type mice (Figure 1C, upper panel). This was also demonstrated by immunohistochemical staining of frozen brain sections (data not shown). To correlate this to total PKA C expression, similar Western blots were analyzed using an anti-PKA C antibody, demonstrating a clear reduction of PKA immunoreactive protein in both Cα KO and Cβ KO animals, as compared to their wild-type and heterozygous littermates (Figure 1C, middle panel). The expression and stability of another EGFR family member, ErbB2, was not affected by PKA depletion (Figure 1C, lower panel), demonstrating specificity of PKA-dependent regulation of the EGFR. EGFR expression levels in heart, kidney, intestine and lung tissue from mice was also analyzed, but due to low EGFR protein levels we were unable to draw conclusions regarding comparison of EGFR protein levels in wt versus knock out animals (data not shown).

Our observation that Cβ KO animals displayed decreased expression of EGFR was unexpected as previous results have indicated that liver mainly expresses the Cα isoform [25]. We therefore examined the total PKA activity in cell extracts of livers from both Cα and Cβ KO animals and their wt littermates using a Kemptide based phosphotransferase assay (Figure 1D). Both Cα and Cβ KO animals demonstrated a similar reduction in total PKA activity of more than 70%, indicating that both Cα and Cβ contribute significantly to total PKA activity in mouse liver.

EGFR expression levels may be regulated at several stages including at the level of transcription, mRNA processing, translation as well as post translation. In order to examine how the EGFR level is regulated, we compared the EGFR mRNA levels in liver of wild type animals with Cα and Cβ KO animals, using real-time PCR.

**Figure 2:**
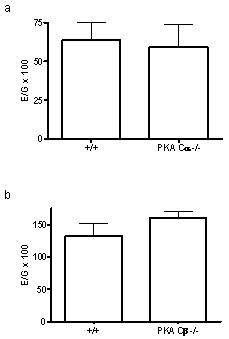
**EGFR mRNA expression in livers from wt, Cα and Cβ KO mice.** The level of EGFR cDNA was determined by real time RT-PCR. The levels of EGFR mRNA expression in Cα KO versus wt livers (a) and Cβ KO versus wt livers (b) were calculated as relative copy numbers normalized against GAPDH mRNA. Relative EGFR mRNA expression was calculated using the formula (E/G) × 100, where E and G are the relative copy numbers of EGFR and GAPDH mRNA, respectively. The results are given as +/- SEM where n = 3.

Deficiency of the PKA Cα or Cβ isoforms did not change expression of EGFR mRNA (Figure 2A,B, respectively). This demonstrated that the reduction in EGFR-levels in Cα and Cβ KO mice livers was not due to reduced mRNA expression, and indicates that a modulation of EGFR at post-transcriptional level is responsible for the reduction in EGFR.

### Ablation of PKA Cα or Cβ reduces the growth in mice

**Figure 3:**
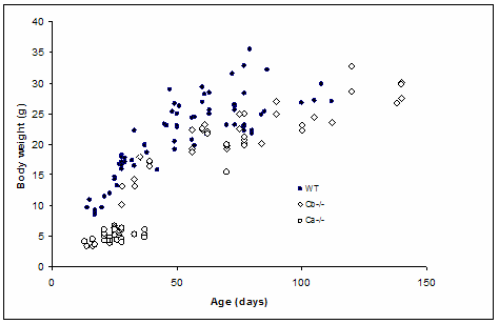
**Postnatal growth of Cα and Cβ KO mice: the figure depicts the correlation between age and measured body weight of WT mice (n = 76) versus Cα KO (n = 32) and Cβ KO (n = 42) mice. **Linear regression analysis showed that both Cα and Cβ KO mice have a significantly lower body weight when compared to WT mice (P < 0,0001) when adjusted for age.

Previously it has been reported that Cα KO mice are growth retarded and thus have significantly reduced body size compared to their wild type littermates [22]. In this study an early postnatal lethality in the majority of Cα KO mice was reported. However, a small percentage of Cα KO mice survived to adulthood [22]. From our preliminary studies with Cα KO mice, we hypothesized that there was a link between body size reduction and a reduced EGFR expression level in these animals. Previous studies did not detect any obvious differences in phenotype of Cβ KO mice [21]. In order to examine if there are any decreases in body size for the Cβ mice, we compared body weight of wt, Cα and Cβ mice at different ages using multiple linear regression in SPSS. As expected, the Cα KO mice were significantly smaller (P < 0.0001) than their wt littermates at all ages (Figure 3). The analysis showed a significant correlation between body weight and genotype when adjusted for age (P < 0.0001). This was also the case with the Cβ KO mice (P < 0.0001). It should however be mentioned that this reduction was much less profound when compared to the Cα KO littermates. Based on this we suggest a redundant function of Cα and Cβ in regulating body size which may be associated with reduced expression of EGFR.

### PKA Cα and Cβ are involved in the regulation of EGFR expression in HeLa cells

**Figure 4:**
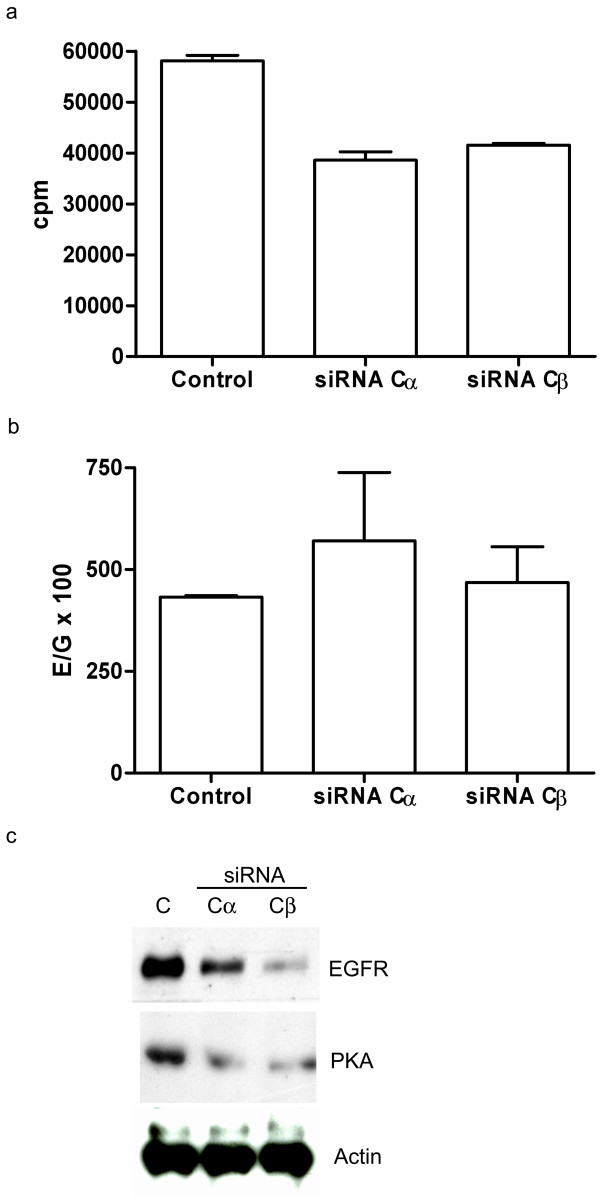
**Levels of EGFR protein correlate with levels and activities of Cα and Cβ in HeLa cells.** (a) HeLa cells incubated with RNAi against Cα and Cβ and monitored for PKA-specific phosphotransferase activity in cell extracts of 1 mg/mL. Relative values given as counts per minute (cpm). (b) Relative EGFR mRNA expression levels were measured by RT-PCR and are given as E/G × 100 where E and G are the relative copy numbers of EGFR and GAPDH mRNA, respectively. The results are given as +/- SEM where n = 3. (c) Western immunoblotting analysis of expression of EGFR and PKA C. An anti-actin antibody was applied to ensure equal loading.

In order to further substantiate the correlation between depletion of PKA catalytic isoforms and expression of the EGFR protein, we studied HeLa cells treated with siRNA directed against Cα and Cβ. Following 24 h incubation with either Cα or Cβ-specific siRNA PKA specific kinase activity was measured by a Kemptide based phosphotransferase assay. This demonstrated a 30% reduction in PKA kinase activity (Figure 4A). When EGFR mRNA levels in the same samples were analyzed by RT-PCR, it was clear that the reduced activity of PKA did not result in a down-regulation of EGFR mRNA (Figure 4B). In contrast the protein level of EGFR was significantly decreased as measured by immunoreactive protein in cell extracts of control cells and cells treated with siRNA against Cα and Cβ (C, Cα and Cβ, respectively, Figure 4C).

## Discussion

The data presented here have demonstrated in two different models that depletion of Cα or Cβ induces a reduction of EGFR protein levels, and that this reduction is not due to decreased level of EGFR mRNA.

A recent report by [10] demonstrated that membrane expression of EGFR was inhibited by the PKA inhibitor H89 through increased internalization and endosome arrest of the EGFR in neuroblastoma N2a cells. Such an effect could be observed after 4 hours of H89 treatment. As H89 is known to have a broader inhibitory specificity [26-28], these observations did not necessarily prove involvement of PKA in maintaining EGFR levels. Here we demonstrate that EGFR was reduced in both mouse liver and HeLa cells after depletion of Cα and Cβ, respectively. The reduced levels of EGFR were not due to reduction in EGFR mRNA, as demonstrated in both HeLa cells depleted of either Cα or Cβ, as well as mouse livers isolated from Cα and Cβ KO mice. The results from mouse Cα and Cβ KO livers indicated a relative reduction in staining for EGFR along the plasma membrane, as well as a general reduction of immunoreactive protein. This is contrary to the previous observation that H89 and thus inhibition of PKA induces an increased internalization of EGFR, which suggests that reduced PKA activity leads to degradation of EGFR. Our results demonstrate in mouse liver as well as HeLa cells, that a reduction in total PKA activity either through elimination of Cα or Cβ leads to a reduced expression of EGFR protein. This was not observed in the brain of neither Cα nor Cβ KO mice. The explanation for this may be that brain has a much higher levels of both PKA Cα and Cβ than liver and HeLa cells [29,15]. Thus, one C subunit may be expected to compensate for the loss of activity of the other isoform. Based on that we postulate that protein levels of EGFR depend on a certain threshold level of PKA and that this effect may not be isoform-specific. Such a hypothesis is in part supported by observations made by Huang and colleagues [30]. In mice with one or two mutant C subunit alleles (Cα -/- and Cβ +/+ or Cα+/+ and Cβ -/- or Cα-/+ and Cβ -/+), either of them were born with apparent embryonic defects. However, when deleting three alleles (either Cα -/- and Cβ -/+, or Cα -/+ and Cβ -/-) they demonstrated a 100% penetrant spinal neural tube defect and spina bifida. This suggests that the C gene dose in the brain is sufficient to rescue a defect irrespective of the isoform, implying Cα and Cβ redundancy in the brain. Furthermore, in our experiments we observed that the regulatory effects on the EGFR levels were apparent even without prior stimulation of endogenous cAMP formation. This observation may be explained in several ways including that of chronic levels of endogenous cAMP, or free C subunits which activity is stimulated independently of cAMP. The latter hypothesis is supported by several studies demonstrating that C subunits when bound to proteins other than the PKA R subunit, the C subunits may be regulated in a cAMP independent fashion [31,32].

The fact that Cβ ablated mice also displayed decreased levels of EGFR indicates that Cβ plays a role in mouse liver, despite previous data indicating low levels of Cβ in mouse liver [15]. This was further substantiated by measurement of PKA activity in mouse liver, demonstrating reduced activity in both Cα and Cβ KO livers. As the sensitivity of Western blotting and mRNA measurements of Cα and Cβ will vary, our measurements of activity in KO mice is probably a better indication of the relative contribution of total C activity from the different isoforms. The reduction in C activity in Cα KO was 70%, while the reduction in C activity in Cβ KO mice was also 70%. This can best be explained by the presence of satiable PKA inhibitors, like endogenous PKI.

The fact that EGFR levels were reduced in the liver of Cβ KO mice is the first observation of a Cβ-dependent phenotype affecting non-nervous tissues. However, we were also able to demonstrate a small but significant reduction in size of the Cβ ablated mice compared to the wt mice, suggesting that the Cβ subunit may influence growth regulation as has been demonstrated for the Cα subunit. The Cα ablated mice showed a growth retardation phenotype consistent with disruption of the GH/IGF-I endocrine system, and while GH-hormone levels were normal, IGF-I mRNA and major urinary proteins (MUPs) levels were reduced, indicating a partial resistance to GH [22]. It has been reported that Cα ablated mice are reduced in size by approximately 30% and approximately 90% die before puberty [22]. Cβ ablated mice by contrast only show a very small reduction in size and they also differ from the Cα mice in that offspring normally survive to adulthood. It is tempting to suggest that Cβ and Cα have redundant effects. Moreover, this may also suggest that cellular signaling pathways involved in growth regulation are merely dependent on a certain level of PKA activity for proper function, rather than isoform-specific effects of Cα and Cβ. As Cα is the most abundant C subunit in non-nervous tissues, disruption of Cα would result in more severe effects in the whole animal than the ablation of the gene for the less abundant Cβ subunit.

High levels of EGFR are associated with many tumors with poor prognostic features. EGFR itself and its downstream signalling pathways are promising targets for anti-tumour drugs [33]. PKA has also been proposed as a possible target for cancer therapy, and the therapeutic potential of the combined blockade of EGFR and PKA has been discussed [34]. With respect to cancer therapy, the focus has been set on the regulatory isoforms of PKA. Site-selective cAMP analogue 8-Cl-cAMP and a series of modified antisense oligonucleotides targeting the PKA RIα subunit have been applied without conclusive effects [35-37]. However, the effects of the cAMP analog 8-Cl-cAMP may be mediated by metabolite rather than the cAMP analog itself, suggesting that PKA is not involved.

## Conclusion

The data presented here have demonstrated in two different models that depletion of Cα or Cβ induces a reduction of EGFR protein levels, and that this reduction is not due to decreased level of EGFR mRNA. Our results have demonstrated that PKA activity contributes to maintaining EGFR levels in cells and in vivo, thus suggesting a cross-talk between the cAMP and EGF pathways, making the PKA C subunit a potential target for future therapies.

## Methods

### Breeding and genotyping of PKA Cα and Cβ null mutated mice

Mice ablated (knock-out, KO) for the Cα, and Cβ genes were kindly contributed by Professor G. Stanley MckKnight (Department of Pharmacology, University of Washington School of Medicine, Seattle, 98195, USA.) [22,21]. All use of animals has been approved and registered by the Norwegian Animal Research Authority. The mice strains were both on a mixed C57BL/6 × 129 background and were treated and bread identically. Animals were housed in a temperature controlled (22°C) facility with a strict 12 hour light/dark cycle, and allowed the RM1 diet (Special Diet Services Ltd, Witham, Essex, UK) and water ad libitum before euthanasia. Heterozygote animals were crossed and offspring genotyped by PCR. The Cα and Cβ KO allele were detected as previously described [22,38].

### Immunofluorescence microscopy

Immunohistochemical analysis of EGFR distribution and expression in liver sections from 6 weeks old wild type and PKA Cα and Cβ KO mice was performed on 8 μm thick sections fixed in ethanol as described previously [39]. Sections were incubated with a sheep anti-EGFR antibody (Fitzgerald) over night, following detection with a Cy2-conjugated donkey anti-sheep IgG antibody (Jackson Immunoresearch). Sections were examined using a Leica TSC XP confocal microscope (Leica Microsystems) equipped with an Ar (488 nm) and two He/Ne (543 and 633 nm) lasers. A Plan apochromat 100×/1.4 oil objective was used.

### Western immunoblotting analysis

Liver and brain from 6 weeks old mice, and HeLa cells, were lysed in Tris lysis buffer, pH 7.4 (50 mM Tris-HCl, 150 mM NaCl, 1 mM EDTA, 1% NP-40, 1 mM Na_3_VO_4_, 20 mM NaF, 1 μg/ml chymostatin, leupeptin and antipain. Liver and brain were homogenized using a Mini beadbeater (Biospec products) with 1 mm silica beads for 20 sec at 4°C. Lysates were incubated on ice for minimum 15 min, and cell debris and nuclei removed by centrifugation at 8000 rpm at 4°C for 10 min. The Dc protein detection kit was applied to measure and adjust total protein concentrations. A 5× protein sample buffer stock solution (2% SDS, 10% glycerol, 0.02% bromophenol blue and 2% β-mercaptoethanol, final concentrations) was mixed with the lysates. The samples were boiled for 5 min, and proteins separated by SDS-PAGE in 6% and 10% gels. Proteins were transferred wet to nitrocellulose membranes, rinsed in ice-cold Tris-buffered saline [TBS; 10 mM Tris, pH 8.0, and 150 mM NaCl] and incubated in blocking buffer (TBS containing 5% dry milk) for 30 min at RT. The membranes were incubated over night at 4°C with donkey anti-EGFR (Fitzgerald), rabbit anti-pan C (anti-PKAαcat, Santa Cruz biotechnology) and mouse anti-PKA C (BD Biosciences), and anti-erbB2 (Zymed). Immunoreactive proteins were recognized using anti-sheep IgG and rabbit IgG conjugated to HRP (Jackson Immunoresearch and Sigma, respectively). Proteins were detected using HRP-conjugated donkey anti-sheep IgG and anti-mouse IgG (Jackson Immunoresearch), and goat anti-rabbit IgG (Sigma) for 90 min at RT. All antibodies were diluted in TBS containing 1% w/v dry milk and 0.01% thimerosal. The filters were washed in TBS, and antigens were visualized by the enhanced chemiluminescence (ECL) method with Hyperfilm MP (Amersham Biosciences).

### Phosphotransferase assay

The PKA kinase activity in liver or HeLa cell was assayed by phosphorylation of the PKA-specific substrate Kemptide (Peninsula Laboratories) using γ-[^32^P]ATP (Amersham Biosciences). The assay was performed in the presence of cAMP. Cells were washed in PBS, harvested by scraping, solubilised in 200 μl homogenising buffer (5 mM KH_2_PO_4_, 5 mM K_2_HPO_4_, 1 mM EDTA, 250 mM sucrose and protease inhibitor cocktail (Roche Diagnostics), pH 6.8) and sonicated for 3 × 5 s. The homogenate was cleared by centrifugation at 16,000 × *g *for 10 min at 4°C. Total protein amount was estimated by Dc protein assay (BioRad). Ten-micro liter homogenate was used in an assay mixture earlier described [40]. Reactions were performed at 30°C, and stopped after 9 min by transfer to P81 phosphocellulose paper (Whatman). Filters were washed in 75 mM phosphorus acid for 1 h at room temperature, incubated with 96% ethanol for 10 min and dried. Activity was measured by liquid scintillation in 3 ml Opti-fluor™ (Packard BioScience).

### RT-PCR

Mouse liver from 6 weeks old mice was homogenized as described above. HeLa cells were washed in PBS, harvested by scraping and subjected to total RNA isolation using the RNeasy Minikit (Qiagen). RNA (1 μg) was used to make first-strand cDNA by Reverse Transcription system (Promega). Titration with cDNA was performed demonstrating that the method was able to detect small changes in sample size. The cDNA was used as templates in PCRs with primer combinations specific for mouse EGFR (5'-TCT TCA AGG ATG TGA AGT GTG-3', 5'-TGT ACG CTT TCG AAC AAT GT-3'). Human EGFR Primers used for EGFR amplification in HeLa cells were: 5'-GCC AAG GCA CGA GTA ACA AGC-3', 5'-AGG GCA ATG AGG ACA TAA CC-3'. The PCR reaction was carried out in a 20 μl final volume containing the following: H_2_O up to 20 μl; 2.4 μl 25 mmol/l MgCl_2_; 1 μl 10 pmol sense primer, 1 μl 10 pmol antisense primer, 2 μl LC-Faststart master mix, and 4 μl cDNA. PCR cycles were as follows: 94°C for 120 s (initial denaturation), 95°C for 5 s, 58°C for 5 s, and 72°C for 13 s (45 cycles).

### Cell culture and RNAi transfection

HeLa cells were cultured in Dulbecco's modified Eagle's medium supplemented with 10% fetal bovine serum (Gibco-BRL), 2 mM l-glutamine and maintained at 37°C and 5% CO_2_. The cells were seeded in 60-mm dishes and all transfections and experiments were initiated two days after plating. Two siRNA duplexes protected by two 3'-overhanged (2'-deoxy) thymidines (dT) were synthesized by Dharmacon Research. These oligonucleotides are: PKA Cα: 5'-AAG CUC CCU UCA UAC CAA AGU-3', PKA Cβ: 5'-AAG GUC CGA UUC CCA UCC CAC-3'. HeLa cells were transiently transfected with siRNA against PKA Cα and Cβ using lipofectamine 2000 (Invitrogen) in Optimem medium (Gibco) at the final concentration of 115 nM. Nontargeting (scrambled) siRNA pool (Dharmacon) at the same concentration was used as a control. After 24 h transfection, cell lysates were collected with Tris lysis buffer and total protein concentration was estimated and adjusted by Dc protein assay (Bio-Rad).

## Authors' contributions

MPO carried out most of the experiments, and participated in the design of the study and writing the manuscript. AF carried out the growth analysis. AKK and HH participated in some of the experiments. ES, HSH, BSS and SØ participated in the design of the study, and writing the manuscript. All authors read and approved the final manuscript.
